# Distribution and clinical role of *KIT* gene mutations in melanoma according to subtype. A study on 492 Spanish patients

**DOI:** 10.1684/ejd.2021.3971

**Published:** 2021-12-01

**Authors:** David Millán-Esteban, Zaida García-Casado, Esperanza Manrique-Silva, Amaya Virós, Rajiv Kumar, Simon Furney, José Antonio López-Guerrero, Celia Requena, Jose Bañuls, Víctor Traves, Eduardo Nagore

**Affiliations:** 1 Laboratory of Molecular Biology. Fundación Instituto Valenciano de Oncología. València, Spain; 2 Department of Dermatology. Fundación Instituto Valenciano de Oncología. València, Spain; 3 Laboratory of Skin Cancer and Aging. CRUK-Manchester Institute. University of Manchester. Manchester. UK; 4 Division of Molecular Genetic Epidemiology, Division of Genomic Functional Analysis. DKFZ. Heidelberg, Germany; 5 Genomic Oncology Research Group, Centre for Systems Medicine, Department of Physiology and Medical Physics, Royal College of Surgeons in Ireland, Dublin, Ireland; 6 Department of Dermatology. Hospital General Universitario de Alicante. Alicante, Spain; 7 Department of Pathology. Fundación Instituto Valenciano de Oncología. València, Spain

**Keywords:** Aggressiveness, KIT, melanoma, mutation, subtype

## Abstract

**Background:**

*KIT* mutations are primarily associated with acral and mucosal melanoma, and have been reported to show higher prevalence in chronic sun-damaged (CSD) than non-CSD melanomas.

**Objectives:**

To investigate the prevalence of *KIT* mutations in melanoma according to subtype, and to determine the clinical role of such mutations.

**Material and Methods:**

Here we present results from a study on a Spanish population of 492 melanomas, classified according to the latest World Health Organization (WHO) guidelines. We analyzed the mutational status of *KIT* and correlated with different clinical variables related to sun exposure and family history.

**Results:**

*KIT* mutations were significantly more frequent in acral (3/36; 8.3%) and mucosal (4/8; 50%) melanomas than in non-acral cutaneous melanomas. No significant difference was observed in *KIT* mutational status between CSD and non-CSD melanomas.

**Conclusion:**

Our results suggested that *KIT* mutations in melanoma tumors are unrelated to nevus proneness or chronic sun damage, but their presence is associated with aggressive melanomas showing ulceration, vascular invasiveness, and increased Breslow thickness. These findings were consistent with those reported by The Cancer Genome Atlas network.

## Introduction

Activating mutations in the gene encoding *KIT* receptor (*KIT*) lead to persistent upregulation of MAPK and PI3K signaling cascades without a ligand (1,2). Several studies have shown mutations in the gene mainly in cutaneous acral and mucosal melanomas in 15-30% of patients. (3–5). As per a divergent pathway hypothesis, melanomas can arise via nevogenic pathway where host factors are critical, and patients present melanocytic instability. In such patients, melanomas are generally located at unexposed or intermittently sun exposed areas of skin without chronic sun damage (CSD). Such lesions usually carry *BRAF* mutations. Alternately, the CSD pathway through cumulative sun exposure leads to melanomas at usually exposed body sites with a substantial degree of solar elastosis that are characterized by a relatively increased proportion of *NRAS* and *KIT* mutations (6–10). Only a few studies have investigated the prevalence of *KIT* mutations in non-acral cutaneous melanoma (11) as most studies have been focused on acral and mucosal melanomas (12,13) (Table 1); In this study, we performed a retrospective investigation on the mutational status of *KIT* gene in primary tumors from a large number of Spanish melanoma patients that included all melanoma subtypes. We determined the association between *KIT* mutations and various clinical variables including different tumor characteristics. Our data showed that the frequency of *KIT* mutations was low in non-acral melanoma and that, contrary to previously reported, prevalence was not different between CSD and non-CSD melanomas. Besides, our evidence suggests the role of *KIT* mutations as an aggressiveness marker.

## Materials and methods

We designed a retrospective study using the melanoma database of the Instituto Valenciano de Oncología (IVO), which contains prospectively collected information from all melanoma patients treated at the institute, a tertiary referral oncology hospital in Spain. The data had been collected from January 2000, included clinical, pathological and epidemiological information assessed by two expert dermatologists at first visit (E. N. and C. R.) (14). The study had the approval of the Ethics Committee at IVO and informed consent from patients.

### Sample recruitment and classification

Melanoma patients included in the study were diagnosed between 2000 and 2018 at IVO. Tumor samples were collected and stored in the Biobank. Melanoma diagnosis was pathologically confirmed by a single pathologist (V. T.). Patients were classified based on the presence/absence of *KIT* mutation. Additionally, patients were classified according to the latest WHO classification of melanomas (non-CSD, CSD, acral and mucosal) (15). In this classification, the difference between non-CSD and CSD was based on the degree of solar elastosis in the unaffected skin surrounding the melanoma.

### DNA extraction and mutation analysis of KIT

DNA was extracted using QIAGEN® commercial kits (QIAamp DNA FFPE Tissue Kit® and QIAamp DNA Investigator Kit®). Exons 9, 11, 13 and 17 of the *KIT* were amplified using primers described in Supplementary Material. PCR was carried using MgCl2 1.5mM; dNTPs 200μM in BufferII 1X; using primers 0.2-0.4μM; and AmpliTaq Gold 1U/tube. The temperature program used for PCR was 95°C-6 min; 40 cycles of 94°C-45 sec, 56°C-1 min, 72°C-1 min; 72°C-10 min. Amplification products were purified and checked in an agarose gel (2%).

Sanger sequencing was performed using 10pM of each primer and the ABI Prism BigDye Terminator Cycle Sequencing kit (Applied Biosystems®). The sequencing conditions were: 96°C-1 min; 25 cycles of 96°C-10 sec, 50°C-5 sec, 60°C-4 min; 4°C hold. Resulting sequencing products were purified via ethanol precipitation and sequenced according to established protocols in a Sanger sequencer (3031x Genetic Analyzer; Applied Biosystems®).

### Statistical analysis

Categorical variables included clinical characteristics and mutational status for *KIT* gene. A *Chi* square test was applied to study differences among the groups, using a threshold of p<0.05 to define statistical significance. First analysis included all selected samples, and the second analysis was carried out by excluding acral and mucosal subtypes (Tables 2 and Supplementary Material).

Melanoma-specific and overall survival were calculated using the Kaplan-Meier method and differences between the curves were tested by log-rank test. The statistical analyses were performed using IBM Corp. Released 2011 (IBM SPSS Statistics for Macintosh, version 20.0. Armonk, NY: IBM Corp.).

## Results

Tumors from 606 melanoma patients with recorded clinical variables were screened for *KIT* mutations. Eleven patients with unknown site of primary tumors were excluded from the analysis. We also excluded 90 patients with no record of peritumoral solar elastosis and 13 patients with rare histological subtypes. Remaining 492 primary tumors from the same number of patients were classified into 4 groups according to the latest WHO guidelines: Non-CSD (384/492; 78.0%), CSD (64/492; 13.0%), acral (36/492; 7.3%) and mucosal (8/492; 1.6%) (Table 2).

*KIT* mutations were present in 4.3% of all tumors (21/492). The highest frequency of *KIT* mutations was in mucosal melanoma (4/8, 50%), followed by acral (3/36, 8.3%), CSD (3/64, 4.7%) and non-CSD melanomas (11/384, 2.9%). The difference in the distribution of KIT mutations in different melanoma subtypes was statistically significant (p<0.001). The distribution of *KIT* mutations in non-cutaneous and cutaneous melanoma is shown in Table 3 and Supplementary Material.

*KIT* mutations were more frequent (p=0.017) in tumors at the sites without sunburns (13/193; 6.7%) than at the sites with either moderate or severe sunburns (5/281; 1.8%). Similarly, the frequency of mutations in melanomas at rarely exposed sites (8/83, 9.6%) was higher (p=0.014) than in tumors at usually or occasionally exposed sites (13/409; 3.2%). The difference in the mutation mutations frequency based on anatomical sites of primary tumors was also statistically significant (p<0.001). The frequency of the mutations in tumors with ulceration was 9.2% (12/130) and 2.5% (9/361) in tumors without ulceration (p=0.004). None of the nevus-associated melanomas had *KIT* mutation, and none of the melanomas harboring *BRAF* mutation carried a *KIT* mutation. No *KIT* mutations were detected in lentigo maligna melanoma (LMM) (Table 2).

We also analyzed the data after exclusion of patients with mucosal and acral melanomas (Supplementary Material). The frequency of *KIT* mutation in all other tumors was 3.1 % (14/448). We observed no statistically significant differences in mutation frequency in tumors from CSD sites (3/64; 4.7%) and non-CSD sites (11/384, 2.9%). The anatomically distribution of the KIT mutations was significantly different (p<0.001) (Supplementary Material).

There was no statistically significant association between *KIT* mutations and family history of melanoma or other cancer type in either entire set of patients or in patients without mucosal and acral melanomas. A trend was observed associating mutations in *KIT* gene with vascular invasion and thicker melanomas (Table 2 and Supplementary Material).

After a median follow-up of 72 months, 108 patients died, 70 of which were due to melanoma. 5-year estimated melanoma-specific and overall survival of patients with KIT mutated melanomas was 77.6% and 73.3%, whereas those without mutations was 82% and 87.5%. Neither comparison showed statistically significant differences (Supplementary Material).

## Discussion

In this study based on the prevalence of *KIT* mutations in melanoma, a large patient set confirmed the high occurrence of such mutations in acral and mucosal melanomas. However, in CSD and non-CSD melanomas, we did not observe the previously reported difference in *KIT* mutation prevalence. This was further corroborated with TCGA data, given that tumors mutated for *KIT* did not associate with signature 7, which is attributed to UV exposure. We also suggested that *KIT* mutations defined aggressiveness in melanoma, which is consistent with TCGA data.

Our results concur with the prevalence of *KIT* mutations in previous studies focused on Caucasian populations as well as with the TCGA population (Beadling et al., 2008; Beretti et al., 2019; Van Engen-Van Grunsven et al., 2014; Yaman, Akalin, & Kandiloʇlu, 2015; Cancer Genome Atlas Network, 2015), but differed in the most prevalent mutated subtype (Supplementary Material). However, it may be pointed out that the number of acral and mucosal melanoma which are generally associated with a high frequency of *KIT* mutations, was low in our dataset.

Our results differ from previous studies reporting a higher prevalence of *KIT* mutations in CSD melanomas (9,10). In fact, we found no significant difference in *KIT* mutation prevalence between CSD and non-CSD tumors. Clinically, melanomas developed at usually sun-exposed skin and with a past history of sunburns were not associated with *KIT* mutations. Interestingly, none of the LMM, the paradigmatic type of chronic sun exposure-associated melanoma, harbored *KIT* mutation. Thus, these findings strengthened the hypothesis that *KIT* mutation are acquired in a CSD-independent manner. Furthermore, we showed that most *KIT* mutated melanomas seemed not to follow a nevogenic development pathway either, given the lower number of nevi in melanoma patients with *KIT* mutated melanomas and that none of the nevus-associated melanomas presented *KIT* mutations. Hence, the development of *KIT* mutations would be independent of the common etiopathogenic pathways.

Also, *KIT* mutations have previously been found to determine a worse melanoma survival (19). In our cohort, survival was worse in *KIT-*mutated melanomas but differences were not statistically significant, most likely due to the small number of *KIT*-mutated cases. However, our pathological results do provide further evidence that links *KIT* mutations and aggressive melanomas because of the association with the presence of ulceration, vascular invasion and increased Breslow thickness in our patients.

This aggressiveness could be expected given the role of *KIT* receptor in the cell. *KIT* mutations lead to the constitutive activation of the receptor, which in turn triggers both MAPK/ERK and PI3K/AKT signaling cascades (9). As a result of their activation, processes such as cell growth and proliferation are enhanced and pro-apoptotic signaling is reduced (20). Moreover, the fact that mutations affect different domains of the protein both extracellular and intracellular makes it more challenging to develop targeted therapies (2).

Our findings were consistent with the information published by the TCGA network. We performed analyses with available sequencing data to check the association between *KIT*. Firstly, concerning the aggressive profile, *KIT* mutations were significantly overrepresented in the “keratin-high” RNA expression group, which was associated with the worst survival. Secondly, the low relation with sun exposure was supported by the fact that *KIT-*mutated melanomas showed a less prevalence of the UV damage-associated Signature 7 (Supplementary Material).

In conclusion, we showed here the so far largest study on *KIT* mutations in melanoma patients for a Spanish population. Our results supported the role of *KIT* mutations as an aggressiveness marker for melanoma patients and suggested that the development of the pathogenesis of *KIT*-mutated melanomas is independent of the common etiopathogenic pathways (nevus-proneness and chronic sun damage).

## Supplementary Material

Supplementary Material

## Figures and Tables

**Table 1:** Prevalence of *KIT* mutations found in literature.

Country	N=Mutated	N=Total	Percentage	Type	Reference
China	223	2793	7.98	All types	Bai et al. (23)
Japan	2	40	5.00	All types	Kaji et al. (24)
Korea	4	47	8.51	Acral	Shim et al. (25)
France	0	20	0	All types	Brocard et al. (26)
China	13	105	12.38	All types	Kang et al. (27)
Brazil	6	29	20.69	Acral Lentiginous Melanoma	De Lima Vazquez et al. (28)
USA	5	15	33.33	Mucosal	Yang et al. (29)
Turkey	4	47	8.51	All types	Yilmaz et al. (30)
Japan	22	171	12.87	All types	Sakaizawa et al. (31)
Turkey	4	106	3.77	All types	Yaman et al. (16)
Netherlands	1	24	4.17	Mucosal	Van Engen-Van et al. (17)
Canada/Germany	7	50	14.00	Mucosal	Aulmann et al. (32)
Italy	4	33	12.12	All types	Massi et al. (33)
China	9	39	23.08	Acral	Dai et al. (34)
Korea	17	202	8.42	All types	Jin et al. (35)
France	1	17	5.88	Mucosal	Chraybi et al. (36)
Sweden	2	56	3.57	Mucosal	Zebary et al. (47)
China	0	20	0	All types	Lin et al. (48)
USA	9	79	11.39	All types	Minor et al. (49)
Spain	5	56	8.93	All types	Botella-Estrada et al. (50)
Japan	3	79	3.80	All types	Ashida et al. (51)
China	7	40	17.50	Mucosal	Ni et al. (37)
USA	42	162	25.93	All types	Carvajal et al. (38)
China	54	502	10.76	All types	Kong et al. (23)
Japan	1	12	8.33	All types	Terada et al. (39)
Japan	1	16	6.25	Mucosal	Sekine et al. (40)
USA	12	189	6.35	All types	Beadling et al. (4)
Germany	5	34	14.71	Mucosal	Satzger et al. (41)
USA	15	101	14.85	All types	Curtin et al. (3)
Italy	1	29	3.45	All types	Beretti et al. (18)
USA	14	122	11.48	Acral	Yeh et al. (42)
USA	1	16	6.25	Mucosal	Lasota (43)
China	15	65	23.08	Mucosal	Zhou et al. (44)
Australia	3	27	11.11	Mucosal	Quek et al. (45)
Mexico	2	62	3.23	Mucosal	Maldonado-Mendoza et al. (46)
Italy	1	69	1.45	All types	Pellegrini et al. (52)
TCGA	12	308	3.90	All types	Cancer Genome Atlas Network 2015
Total	**527**	**5782**	**9.11**		

**Table 2:** Distribution of melanomas tested for *KIT* among clinical variables.

	Total		cKIT				
Variable			Wild type		Mutated		p value
	N	%	N	%	N	%	
**WHO Groups**							
*Non-CSD*	384	78.0	373	97.1	11	2.9	<0.001
*CSD*	64	13.0	61	95.3	3	4.7	
*Acral*	36	7.3	33	91.7	3	8.3	
*Mucosal*	8	1.6	4	50	4	50	
**Sex**							
*Men*	257	52.2	249	96.9	8	3.1	0.264
*Women*	235	47.8	222	94.5	13	5.5	
**Sunburns in MM area**							
*Absent*	193	40.7	180	93.3	13	6.7	0.017
*Weak/moderate*	172	36.3	170	98.8	2	1.2	
*Severe*	109	23.0	106	97.2	3	2.8	
**Past personal history of severe sunburns**							
≤ *5*	402	83.2	387	96.3	15	3.7	1
>*5*	81	16.8	78	96.3	3	3.7	
**Solar Lentigos**							
*No*	65	13.9	61	93.8	4	6.2	0.274
*Yes*	404	86.1	391	96.8	13	3.2	
**Solar Lentigos at MM area**							
*No*	281	58.5	265	94.3	16	5.7	0.114
*Yes*	199	41.5	194	97.5	5	2.5	
**Second tumor**							
*No*	422	85.9	403	95.5	19	4.5	0.335
*Yes*	69	14.1	68	98.6	1	1.4	
**History of non-melanoma skin cancer**							
*No*	454	92.3	435	95.8	19	4.2	0.672
*Yes*	38	7.7	36	94.7	2	5.3	
**Number of nevi**							
<*20*	318	66.1	301	94.7	17	5.3	0.027
≥*20*	163	33.9	161	98.8	2	1.2	
**Multiple melanoma**							
*No*	470	95.9	452	96.2	18	3.8	0.048
*Yes*	20	4.1	17	85.0	3	15.0	
**Family history of melanoma**							
*No*	458	93.7	439	95.9	19	4.1	0.635
*Yes*	31	6.3	29	93.5	2	6.5	
**Family history of pancreatic cancer**							
*No*	465	95.1	445	95.7	20	4.3	1
*Yes*	24	4.9	23	95.8	1	4.2	
**Family history of cancer**							
*No*	241	49.3	234	97.1	7	2.9	0.181
*Yes*	248	50.7	234	94.4	14	5.6	
**Sun exposure pattern in MM area**							
*Rare*	83	16.9	75	90.4	8	9.6	0.014
*Occasional*	317	64.4	309	97.5	8	2.5	
*Usual*	92	18.7	87	94.6	5	5.4	
**Anatomic site of the primary**							
*Head and Neck*	93	18.9	91	97.8	2	2.2	<0.001
*Upper Limbs*	67	13.6	65	97.0	2	3.0	
*Trunk*	182	37.0	179	98.4	3	1.6	
*Lower Limbs*	78	15.9	75	96.2	3	3.8	
*Acral*	64	13.0	57	89.1	7	10.9	
*Mucosal*	8	1.6	4	50.0	4	50.0	
**Histological type**							
*LMM*	27	5.5	27	100.0	0	0	<0.001
*SSM*	305	62.0	294	96.4	11	3.6	
*NM*	119	24.2	116	97.5	3	2.5	
*ALM*	36	7.3	33	91.7	3	8.3	
*Others*	5	1.0	1	20.0	4	80.0	
**Ulceration**							
*Absence*	361	73.5	352	97.5	9	2.5	0.004
*Presence*	130	26.5	118	90.8	12	9.2	
**Microscopic satellite**							
*No*	466	95.1	446	95.7	20	4.3	1
*Yes*	24	4.9	23	95.8	1	4.2	
**Vascular invasion**							
*No*	475	97.5	456	96.0	19	4.0	0.09
*Yes*	12	2.5	10	83.3	2	16.7	
**Associated nevus**							
*No*	380	78.0	360	94.7	20	5.3	0.011
*Yes*	107	22.0	107	100.0	0	0	
**CSD**							
*Non-CSD*	428	87.0	410	95.8	18	4.2	0.745
*CSD*	64	13.0	61	95.3	3	4.7	
**Stage**							
*In situ*	19	3.9	19	100.0	0	0	0.389
*Localized*	341	69.3	329	96.5	12	3.5	
*Locoregional*	126	25.6	117	92.9	9	7.1	
*Distant*	5	1.0	5	100.0	0	0	
*Unknown*	1	0.2	1	100.0	0	0	
**BRAF**							
*WT*	280	57.3	259	92.5	21	7.5	<0.001
*Mutated*	209	42.7	209	100.0	0	0	
**NRAS**							
*WT*	445	90.8	426	95.7	19	4.3	0.949
*Mutated*	43	8.8	41	95.3	2	4.7	
*Unknown*	2	0.4	2	100.0	0	0	
**Breslow thicknes**							
*≤2 mm*	286	60.5	277	96.9	9	3.1	0.111
*>2 mm*	187	39.5	175	93.6	12	6.4	

**Table 3:** Distribution of *KIT* mutations within our cohort.

N° of cases	%	WHO Classification	Exon	Nucleotide change	Protein change
			(NM_000222.2)		
**5**	23.8	1 mucosal, 2 non-CSD, 2 acral	11	c.1727T>C	p.(L576P)
**3**	14.3	1 mucosal, 1 non-CSD, 1 CSD	11	c.1924A>G	p.(K642E)
**1**	4.8	Non-CSD	11	c.1676T>A	p.(V559D)
**1**	4.8	Non-CSD	11	c.1676T>C	p.(V559A)
**1**	4.8	Non-CSD	11	c.1660_1674del	p.(E554_K558del)
**1**	4.8	Non-CSD	13	c.1936_1937delTA	p.(Y646Pfs*3)
**1**	4.8	CSD	11	c.1735G>A	p.(D579N)
**1**	4.8	Acral	11	c.1729_1734dup	p.(P577_Y578dup)
**1**	4.8	Non-CSD	11	c.1655_1672del18	p.M552_W557del
**1**	4.8	Mucosal	9	c.1463C>A	p.(T488K)
**1**	4.8	CSD	11	c.1732_1734delTAT	p.(Y578del)
**1**	4.8	Non-CSD	17	c.2458G>T	p.(D820Y)
**1**	4.8	Mucosal	13	c.1936T>G	p.(Y646D)
**1**	4.8	Non-CSD	9	c.1463C>T	p.T488M
**1**	4.8	Non-CSD	9	c.1427G>T; c.1430C>T	p.(S476I); p.(S477F)
