# Supplementary material for: Distribution and clinical role of KIT gene mutations in melanoma according to subtype. A study on 492 Spanish patients
Source: Eur J Dermatol. Author manuscript; Available in PMC 2023 Sep 6. (PMC7615026; doi:10.1684/ejd.2021.3971)
Supplement: Supplementary Material [file EMS185041-supplement-Supplementary_Material.docx]

*Definition of clinical variables*

-Sunburns in melanoma (MM) area: Personal history of sunburn at the area where the melanoma is presented, according to the patient’s memories. Severe category includes those producing blisters whereas weak/moderate category includes those not producing blisters.

-Past personal history of severe sunburns: Number of lifetime severe sunburns (defined as those associated to blisters), as recalled by the patients.

-Solar lentigos: Clinical presence of solar lentigines at any part of the body, determined by dermatological examination at first visit.

-Solar lentigos at melanoma (MM) area: Clinical presence of solar lentigos at the skin area surrounding the melanoma, as determined by dermatological examination at first visit.

-Second non-cutaneous neoplasia: Past personal history of other non-cutaneous neoplasia.

-History of non-melanoma skin cancer: Past personal history of non-melanoma skin cancer (accounting for CBC or SCC).

-Number of nevi: Total number of common melanocytic nevus with > 2 mm, excluding scalp and the genital area, determined at the physical examination by the dermatologist at first visit.

-Multiple melanoma: Personal history of more than one diagnosed melanoma.

-Family history of melanoma: Presence of at least one first- or second-degree relative with personal history of melanoma.

-Family history of pancreatic cancer: Presence of at least one first- or second-degree relative with personal history of pancreatic cancer.

-Family history of cancer: Presence of at least one first-degree relative with personal history of other non-cutaneous cancer.

-Sun exposure pattern of MM area: Defined by the relative frequency the cutaneous area where the melanoma develops had been exposed to the sun, according to the patient’s provided information. The categories do not assume any particular pattern per se but according to the patient’s particular behavior. Categories are defined as follows: ‘Rarely’ accounts for areas that almost always covered by clothes or abundant hair (i.e. non-bald scalp); ‘Occasionally’ includes areas that are most of the time covered but exposed in summer or sometimes during the rest of the year; ‘Usually’ refers to those areas that are exposed frequently to the sun because of type of work (outdoor) or frequent recreational activities during the year.

-Anatomic site of the primary.

-Clinical-pathological subtype: categorized into lentigo maligna melanoma (LMM), superficial spreading melanoma (SSM), nodular melanoma (NM), acral lentiginous melanoma (ALM), and other subtypes or not otherwise specified (NOS).

-Ulceration: histologically determined by the pathologist and excluding trauma-induced ulceration.

-Microscopic satellite: Presence of one or more discontinuous nests of neoplastic melanocytes clearly separated by normal dermis from the main bulk of the melanoma, determined by pathological examination.

-Vascular invasion: presence of unequivocal melanoma cells, in nests or isolated, in the lumen of dermal vessels, histologically assessed by the pathologist.

-Associated nevus: Presence or absence of remnants of a melanocytic nevus (congenital, common, or dysplastic) adjacent to the melanoma, determined by the pathologist.

-CSD: Histological presence or absence of different degree of solar elastosis in the unaffected skin surrounding the melanoma, determined by the pathologist.

-Stage: After pathological staging, including clinical examination, image tests and sentinel lymph node biopsy, when indicated.

-Breslow thickness: histologically criteria determined by the pathologist and categorized for this study into categories: T1-T2 vs. T3-T4 (using current TNM categories).

# Supplementary Table 1. KIT primer sequences

| **Exon** | **Primer** | **Primer sequence** |
| --- | --- | --- |
| **9** | Forward | CTTCCCTTTAGATGCTCTGCTTCTG |
|  | Reverse | CAGAGCCTAAACATCCCCTTAAATTGG |
| **11** | Forward | CTCTCTCCAGAGTGCTCTAATGAC |
|  | Reverse | GTTCCTTAAAGTCACTGTTATCTCTACC |
| **13** | Forward | GACATCAGTTTGCCAGTTGTGC |
|  | Reverse | CCAAGCAGTTTATAATCTTAGCATTGCC |
| **17** | Forward | AAATGGTTTTCTTTTCTCCTCCAACC |
|  | Reverse | TCCTTTGCAGGACTGTCAAGC |

1

# Supplementary Table 2. Distribution of CSD and non-CSD melanomas tested for KIT among clinical variables.

| **Variable** | **Total** | | **cKIT** | | | | **p value** |
| --- | --- | --- | --- | --- | --- | --- | --- |
|  |  |  | **Wild type** | | **Mutated** | |  |
|  | **N** | **%** | **N** | **%** | **N** | **%** |  |
| **Sex** |  |  |  |  |  |  |  |
| *Men* | 240 | 53.6 | 234 | 97.5 | 6 | 2.5 | 0.430 |
| *Women* | 208 | 46,4 | 200 | 96.2 | 8 | 3.8 |  |
| **Sunburns in MM area** |  |  |  |  |  |  |  |
| *Absent* | 155 | 35.7 | 148 | 95.5 | 7 | 4.5 | 0.186 |
| *Weak/moderate* | 170 | 39.2 | 168 | 98.8 | 2 | 1.2 |  |
| *Severe* | 109 | 25.1 | 106 | 97.2 | 3 | 2.8 |  |
| **Past personal history of severe sunburns** |  |  |  |  |  |  |  |
| *≤5* | 365 | 82.8 | 355 | 97.3 | 10 | 2.7 | 0.476 |
| *>5* | 76 | 17.2 | 73 | 96.1 | 3 | 3.9 |  |
| **Solar Lentigos** |  |  |  |  |  |  |  |
| *No* | 55 | 12.9 | 55 | 100.0 | 0 | 0 | 0.378 |
| *Yes* | 373 | 87.1 | 361 | 96.8 | 12 | 3.2 |  |
| **Solar Lentigos at MM area** |  |  |  |  |  |  |  |
| *No* | 239 | 54.7 | 230 | 96.2 | 9 | 3.8 | 0.589 |
| *Yes* | 198 | 45.3 | 193 | 97.5 | 5 | 2.5 |  |
| **Second tumor** |  |  |  |  |  |  |  |
| *No* | 383 | 85.5 | 370 | 96.6 | 13 | 3.4 | 0.703 |
| *Yes* | 65 | 14.5 | 64 | 98.5 | 1 | 1.5 |  |
| **Personal history of non-melanoma skin cancer** |  |  |  |  |  |  |  |
| *No* | 412 | 92.0 | 400 | 97.1 | 12 | 2.9 | 0.312 |
| *Yes* | 36 | 8.0 | 34 | 94.4 | 2 | 5.6 |  |
| **Number of nevi** |  |  |  |  |  |  |  |
| *<20* | 282 | 63.9 | 270 | 95.7 | 12 | 4.3 | 0.097 |
| *≥20* | 159 | 36.1 | 157 | 98.7 | 2 | 1.3 |  |
| **Multiple melanoma** |  |  |  |  |  |  |  |
| *No* | 428 | 96.0 | 416 | 97.2 | 12 | 2.8 | 0.105 |
| *Yes* | 18 | 4.0 | 16 | 88.9 | 2 | 11.1 |  |
| **Family history of melanoma** |  |  |  |  |  |  |  |
| *No* | 414 | 93.0 | 402 | 97.1 | 12 | 2.9 | 0.254 |
| *Yes* | 31 | 7.0 | 29 | 93.5 | 2 | 6.5 |  |
| **Family history of pancreatic cancer** |  |  |  |  |  |  |  |
| *No* | 422 | 94.8 | 409 | 96.9 | 13 | 3.1 | 0.530 |
| *Yes* | 23 | 5.2 | 22 | 95.7 | 1 | 4.3 |  |
| **Family history of cancer** |  |  |  |  |  |  |  |
| *No* | 223 | 50.1 | 218 | 97.8 | 5 | 2.2 | 0.293 |
| *Yes* | 222 | 49.9 | 213 | 95.9 | 9 | 4.1 |  |
| **Sun exposure pattern in MM area** |  |  |  |  |  |  |  |
| *Rarely* | 49 | 10.9 | 47 | 95.9 | 2 | 4.1 | 0.578 |
| *Occasionaly* | 312 | 69.6 | 304 | 97.4 | 8 | 2.6 |  |
| *Usually* | 87 | 19.4 | 83 | 95.4 | 4 | 4.6 |  |
| **Anatomic site of the primary** |  |  |  |  |  |  |  |
| *Head and Neck* | 93 | 22.2 | 91 | 97.8 | 2 | 2.2 | <0.001 |
| *Upper Limbs* | 66 | 15.8 | 65 | 98.5 | 1 | 1.5 |  |
| *Trunk* | 182 | 43.4 | 179 | 98.4 | 3 | 1.6 |  |

1

| *Lower Limbs* | 78 | 18.6 | 75 | 96.2 | 3 | 3.8 |  |
| --- | --- | --- | --- | --- | --- | --- | --- |
| **Histological type** |  |  |  |  |  |  |  |
| *LMM* | 27 | 6.0 | 27 | 100.0 | 0 | 0 | 0.539 |
| *SSM* | 304 | 67.9 | 293 | 96.4 | 11 | 3.6 |  |
| *NM* | 117 | 26.1 | 114 | 97.4 | 3 | 2.6 |  |
| **Ulceration** |  |  |  |  |  |  |  |
| *Absence* | 340 | 76.1 | 331 | 97.4 | 9 | 2.6 | 0.339 |
| *Presence* | 107 | 23.9 | 102 | 95.3 | 5 | 4.7 |  |
| **Microscopic satellite** |  |  |  |  |  |  |  |
| *No* | 424 | 94.9 | 410 | 96.7 | 14 | 3.3 | 1 |
| *Yes* | 23 | 5.1 | 23 | 100.0 | 0 | 0 |  |
| **Vascular invasion** |  |  |  |  |  |  |  |
| *No* | 433 | 97.5 | 420 | 97.0 | 13 | 3.0 | 0.300 |
| *Yes* | 11 | 2.5 | 10 | 90.9 | 1 | 9.1 |  |
| **Associated nevus** |  |  |  |  |  |  |  |
| *No* | 338 | 76.3 | 325 | 96.2 | 13 | 3.8 | 0.045 |
| *Yes* | 105 | 23.7 | 105 | 100.0 | 0 | 0 |  |
| **CSD** |  |  |  |  |  |  |  |
| *Non-CSD* | 384 | 85.7 | 373 | 97.1 | 11 | 2.9 | 0.434 |
| *CSD* | 64 | 14.3 | 61 | 95.3 | 3 | 4.7 |  |
| **Stage** |  |  |  |  |  |  |  |
| *In situ* | 12 | 2.7 | 12 | 100.0 | 0 | 0 | 0.961 |
| *Localized* | 319 | 71.2 | 309 | 96.9 | 10 | 3.1 |  |
| *Locoregional* | 112 | 25.0 | 108 | 96.4 | 4 | 3.6 |  |
| *Distant* | 4 | 0.9 | 4 | 100.0 | 0 | 0 |  |
| *Unknown* | 1 | 0.2 | 1 | 100.0 | 0 | 0 |  |
| **BRAF** |  |  |  |  |  |  |  |
| *WT* | 242 | 54.4 | 228 | 94.2 | 14 | 5.8 | <0.001 |
| *Mutated* | 203 | 45.6 | 203 | 100.0 | 0 | 0 |  |
| **NRAS** |  |  |  |  |  |  |  |
| *WT* | 407 | 91.3 | 394 | 96.8 | 13 | 3.2 | 0.955 |
| *Mutated* | 37 | 8.3 | 36 | 97.3 | 1 | 2.7 |  |
| *Unknown* | 2 | 0.4 | 2 | 100.0 | 0 | 0 |  |
| **Breslow thickness** |  |  |  |  |  |  |  |
| *≤2 mm* | 269 | 61.7 | 261 | 97.0 | 8 | 3.0 | 0.783 |
| *>2 mm* | 167 | 38.3 | 161 | 96.4 | 6 | 3.6 |  |

2

**Supplementary Table 3. Detailed information on KIT mutated cases within our cohort.**

| **Identification** | **Age** | **Sex** | **WHO Classification** | **Localization** | **Histological Type** | **Ulceration** | **Breslow** | **KIT status (NM_000222.2)** | | |
| --- | --- | --- | --- | --- | --- | --- | --- | --- | --- | --- |
|  |  |  |  |  |  |  |  | **Exon** | **Nucleotide change** | **Protein change** |
| **80** | 42 | Male | Non-CSD | Back | NM | Yes | 3.3 | 11 | c.1660_1674del | p.(E554_K558del) |
| **117** | 49 | Male | Mucosal | Glans penis | Mucosal | Yes | 9.0 | 11 | c.1727T>C | p.(L576P) |
| **157** | 59 | Female | Non-CSD | Sole | NM | Yes | 5.5 | 11 | c.1727T>C | p.(L576P) |
| **417** | 61 | Male | Acral | Sole | ALM | Yes | 12.0 | 11 | c.1727T>C | p.(L576P) |
| **740** | 60 | Female | Non-CSD | Foot dorsum | SSM | No | 1.2 | 13 | c.1936_1937delTA | p.(Y646Pfs*3) |
| **1494** | 61 | Male | Non-CSD | Left palm | SSM | Yes | 5.0 | 11 | c.1924A>G | p.(K642E) |
| **1663** | 53 | Female | CSD | Left temple | SSM | No | 0.28 | 11 | c.1735G>A | p.(D579N) |
| **1776** | 88 | Male | CSD | Left forearm | SSM | No | 1.10 | 11 | c.1924A>G | p.(K642E) |
| **1817** | 48 | Female | Acral | Subungueal left thumb | ALM | Yes | 5.5 | 11 | c.1729_1734dup | p.(P577_Y578dup) |
| **1868** | 79 | Female | Non-CSD | Left Calf | SSM | No | 1.75 | 11 | c.1727T>C | p.(L576P) |
| **1948** | 35 | Female | Non-CSD | Right Thigh | SSM | No | 1.0 | 11 | c.1676T>A | p.(V559D) |
| **2056** | 84 | Female | Acral | Subungueal left thumb | ALM | Yes | 1.16 | 11 | c.1727T>C | p.(L576P) |
| **2062** | 38 | Female | Non-CSD | Arch of the foot | SSM | No | 0.7 | 11 | c.1655_1672del18 | p.M552_W557del |
| **2081** | 73 | Female | Mucosal | Perianal | Mucosal | Yes | 5.8 | 9 | c.1463C>A | p.(T488K) |
| **2112** | 53 | Female | CSD | Thorax | SSM | Yes | 0.81 | 11 | c.1732_1734delTAT | p.(Y578del) |
| **2131** | 61 | Male | Non-CSD | Sole | SSM | Yes | 4.9 | 17 | c.2458G>T | p.(D820Y) |
| **2167** | 73 | Female | Mucosal | Vulvar | Unclassified | Yes | 8.0 | 13 | c.1936T>G | p.(Y646D) |
| **2403** | 82 | Male | Non-CSD | Scalp (bald) | SSM | No | 2.45 | 9 | c.1463C>T | p.T488M |
| **2434** | 75 | Male | Non-CSD | Back | SSM | No | 0.4 | 11 | c.1676T>C | p.(V559A) |
| **2451** | 76 | Female | Non-CSD | Leg | NM | No | 6.9 | 9 | c.1427G>T; c.1430C>T | p.(S476I); p.(S477F) |
| **2482** | 86 | Female | Mucosal | Vulvar | Mucosal | Yes | 23.0 | 11 | c.1924A>G | p.(K642E) |

1

# Supplementary Table 4. KIT mutation prevalence according to subtype and race.

| **Type of melanoma** | **N mutated** | **N wild-type** | **Percentage** |
| --- | --- | --- | --- |
| *Cutaneous* | 432 | 4815 | 8,23 |
| *Mucosal* | 50 | 422 | 10,59 |
| *Acral* | 33 | 237 | 12,22 |
| **Race distribution** |  |  |  |
| ***Caucasian*** | 143 | 1274 | 10,09 |
| *Cutaneous* | 97 | 891 | 9,82 |
| *Mucosal* | 27 | 301 | 8,23 |
| *Acral* | 20 | 151 | 11,70 |
| ***Asian*** | 371 | 4131 | 8,24 |
| *Cutaneous* | 335 | 3924 | 7,87 |
| *Mucosal* | 23 | 121 | 15,97 |
| *Acral* | 13 | 86 | 13,13 |

*** TCGA not included

1

**Supplementary Material.**

The Cancer Genome Atlas (TCGA) network reported a complete genomic profile for cutaneous melanoma (11) that included mutational status of *KIT*. Mutations in *KIT* were only present in 12 (3.5%) of the melanomas. Besides, a transcriptomic classification of melanoma into three groups (“immune-high”, “keratin-high” and “*MITF*-low”), based on consensus hierarchical clustering analysis was proposed. Patient survival differed significantly among the groups but difference in prevalence based on mutational status had not been performed.

*The Cancer Genome Atlas (TCGA) data analysis*

For the TCGA study, data from the whole available set were downloaded from TableS1 at

*https://*[*www.sciencedirect.com/science/article/pii/S0092867415006340?via%3Dihub#app2*.](http://www.sciencedirect.com/science/article/pii/S0092867415006340?via%3Dihub&amp;app2) We

established a *Signature 7* status, based on DNA damage due to UV radiation, for all patients. This signature was described based on trinucleotide combinations. Signature 7 was characterized, mostly but not exclusively, by C>T changes in a dipyrimidine context at the first two nucleotides (21, 22). For the TCGA mutation data signature analysis, the primary data were the somatic mutation calls from TCGA MAF (skcm_clean_pairs.aggregated.capture.tcga.uuid.somatic.maf) of the whole-exome sequences of tumors from the TCGA skin cutaneous melanoma (SKCM) cohort. Mutational signature analysis was conducted using the R package deconstructSigs (53) to estimate the proportion of Signature 7 mutations in each tumor genome (*https://cancer.sanger.ac.uk/cosmic/signatures_v2*). For a patient classified as Signature 7 positive had at least 25% of the mutations as per the signature (22). The expression cluster was also extracted for the TCGA cohort, in which tumors had been clustered in “immune-high”, “keratin-high” and “MITF-low” (11).

The analysis of the TCGA data showed that the frequency of the “keratin-high” expression pattern tumors was higher in *KIT* mutated melanomas 9/12 (75%) than in wild type 83/304 (27.3%); the

frequency of “immune” tumors was lower in mutated melanomas 2/12 (16.7%) than in wild type 163/304 (53.6%); and the frequency of “*MITF-*low” was lower in mutated melanomas 1/12 (8.3%) than in wild type 58/304 (19.1%) (p=0.004).

We also found a lower proportion of Signature 7 cases within *KIT* mutated melanomas (7/12; 58.3%) than in *KIT* wild type (233/308; 75.6%) albeit without statistical significance (p=0.183).

**
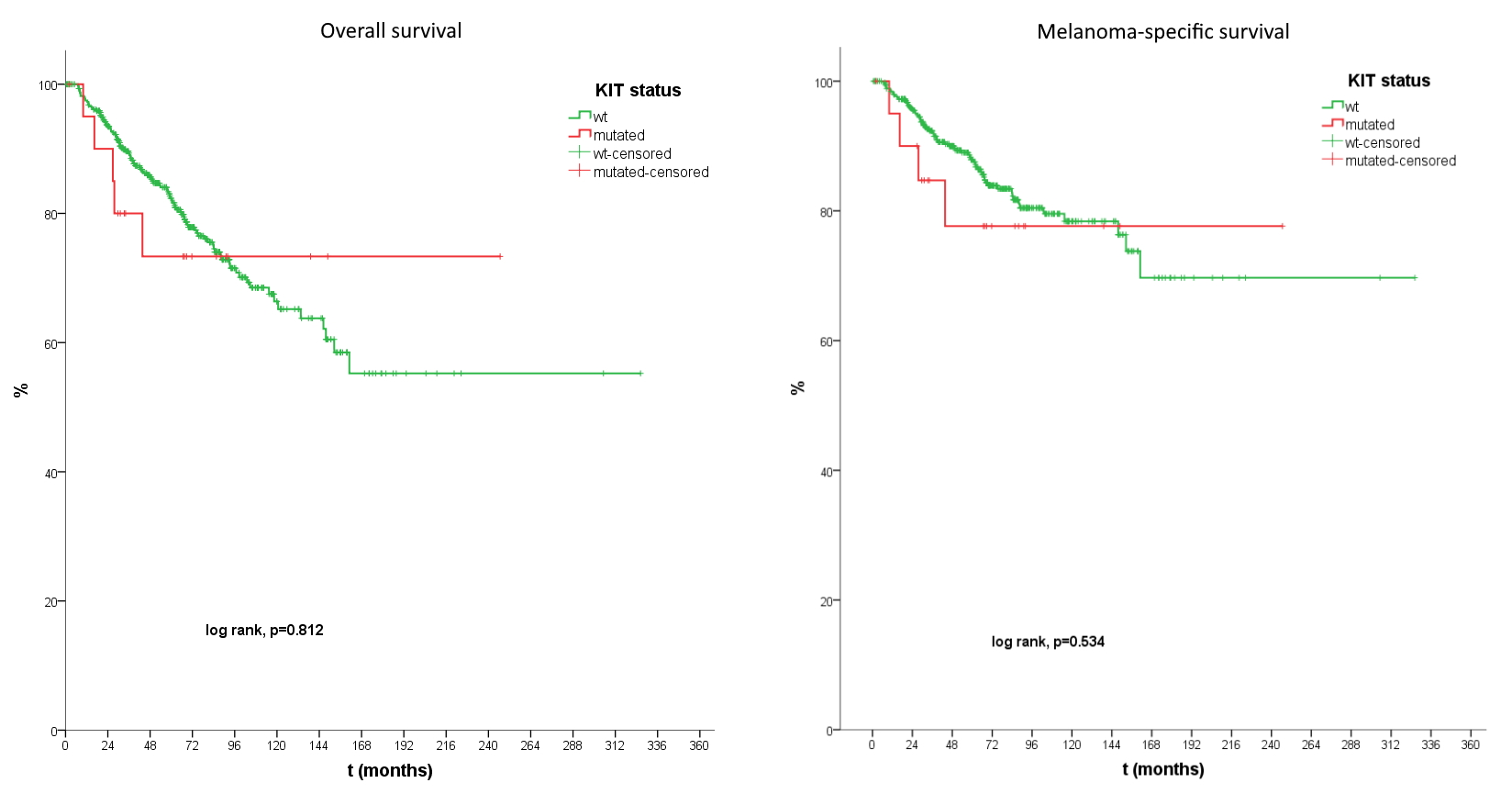
Supplementary Figure. Survival charts.**
